# A first manic episode in an elderly patient

**DOI:** 10.1192/j.eurpsy.2021.1644

**Published:** 2021-08-13

**Authors:** E. Rodríguez Vázquez, C. Capella Meseguer, G. Guerra Valera, A. Gonzaga Ramírez, M. Queipo De Llano De La Viuda, I. Santos Carrasco, J. Gonçalves Cerejeira

**Affiliations:** 1 Psiquiatría, Hospital Clínico Universitario de Valladolid, Valladolid, Spain; 2 Psiquiatría, HCUV, Valladolid, Spain

**Keywords:** bipolar disorder, elder patient, first episode, manic

## Abstract

**Introduction:**

The bipolar disorder is characterized by instability mood. It normally happens in the middle-aged. In elderly patients with a first manic episode, you have to dismiss organic pathology.

**Objectives:**

To present an elderly patient with a first manic episode

**Methods:**

A descriptive study of a clinical case and literature review

**Results:**

A 67-year-old man, married. Consulted Mental Health 5 years ago, about low mood after his early retirement. With no psychiatric treatment. Somatic antecedents: Acute myocardial infarction, hypertension and dyslipidemia. Came to the hospital accompanied by his son-in-law presenting rapid speech and thinking, bright clothing, risky behaviour, irritability, grandiosity deliration ideas and less sleep; being necessary a hospital admission. Blood and urine analysis: with no abnormalities. No toxics in the urine. Asenapine 20mg and Lorazepam 3mg were prescribed with clinical improvement. Brain CT: with no abnormalities. After that, Lithium 400mg per day was prescribed to avoid the induction of depression. Clinical judgement: manic episode. Bipolar disorder Type I

**Conclusions:**

After dismissing somatic causes, the symptomatic treatment of a manic episode in older patients is on the same lines as the treatment for mania in young adults. 8-10% of psychiatric inpatients over age 55-60 years are diagnosed with bipolar disorder. Since there is an increase in the number of individuals living longer, an expected increase in the number of older adults who will be diagnosed with bipolar disorder. Older adults with bipolar disorder will increase in absolute numbers as well as the proportion of the general populations.

**Disclosure:**

No significant relationships.

